# Phenotypic switching of vascular smooth muscle cells: a central mechanism in vein graft intimal hyperplasia

**DOI:** 10.3389/fcvm.2025.1713297

**Published:** 2026-01-09

**Authors:** Linyuan Wang, Yongzhi Deng

**Affiliations:** 1 Department of Cardiovascular Surgery, The Affiliated Hospital of Shanxi Medical University, Shanxi Cardiovascular Hospital, Shanxi Clinical Medical Research Center for Cardiovascular Disease, Taiyuan, China; 2 Department of Cardiovascular Surgery, Shanxi Cardiovascular Hospital, The Affiliated Hospital of Shanxi Medical University, Shanxi Clinical Medical Research Center for Cardiovascular Disease, Taiyuan, China

**Keywords:** coronary artery bypass grafting, intimal hyperplasia, phenotypic switching, vascular smooth muscle cells, vein graft failure

## Abstract

Coronary artery bypass grafting (CABG) remains the cornerstone of revascularization for patients with complex coronary artery disease. While the great saphenous vein (GSV) is the most widely used conduit, its long-term patency is limited by postoperative intimal hyperplasia (IH) and accelerated atherosclerosis. Central to this pathological process is the phenotypic switching of vascular smooth muscle cells (VSMCs) from a quiescent contractile state to a proliferative, migratory, and synthetic phenotype. This review systematically summarizes the structural and functional differences between venous and arterial grafts, the sequential pathological mechanisms of vein graft failure, and the molecular drivers of VSMCs phenotypic switching. Key regulatory pathways—including PDGF-BB, TGF-β, MAPK, mTOR, and NF-κB—as well as non-coding RNAs, orchestrate this process in response to endothelial dysfunction, inflammatory activation, and altered hemodynamics. In addition, emerging therapeutic strategies aimed at mitigating IH are discussed, including optimized surgical harvesting techniques, improved conduit preservation solutions, pharmacological agents, gene therapy, and venous external stenting. Despite significant advances, the complexity of VSMCs regulatory networks and the limitations of current interventions underscore the need for integrative approaches combining molecular targeting with innovative delivery systems. Elucidating these mechanisms holds promise for enhancing long-term vein graft patency and improving outcomes in patients undergoing CABG.

## 1 Introduction

With the advancement of the social economy and the aging population, the global burden of coronary artery disease (CAD) has significantly increased, making it a major public health concern (1). For patients with complex CAD—such as multivessel disease and/or left main coronary artery disease—coronary artery bypass grafting (CABG) remains the gold standard for revascularization and plays a critical role in clinical practice (2).

Commonly used vascular grafts in CABG include the left internal mammary artery (LIMA), radial artery (RA), and great saphenous vein (GSV). Although arterial grafts offer superior long-term patency, their clinical use is often limited by anatomical constraints, such as insufficient vessel length or donor vessel stenosis (3). In contrast, the GSV is easily accessible and sufficiently long, making it the most widely used venous conduit in CABG (4, 5). However, GSV grafts exhibit a relatively high failure rate—approximately 40%–50% within 10 years post-surgery—which seriously compromises long-term graft patency and patient prognosis (6, 7). This high failure rate is primarily attributed to vein graft disease (VGD), characterized by pronounced intimal hyperplasia (IH) and accelerated atherosclerosis, ultimately leading to graft occlusion and recurrent ischemic symptoms (8).

Among the pathogenic mechanisms of VGD, the phenotypic switching of vascular smooth muscle cells (VSMCs) plays a central role (9). Under physiological conditions, VSMCs exhibit a contractile phenotype, marked by high expression of contractile proteins such as α-smooth muscle actin (α-SMA) and smooth muscle 22α (SM22α) (10). Following CABG, VSMCs are exposed to pathological stimuli—including inflammatory cytokines, oxidative stress, and hemodynamic changes—which induce their transformation into a synthetic phenotype (11). This switch is characterized by downregulation of contractile markers, enhanced proliferative and migratory capacity, and increased secretion of extracellular matrix (ECM) components and pro-inflammatory mediators, all of which promote IH development (12). Therefore, elucidating the regulatory mechanisms underlying VSMCs phenotypic switching is of great theoretical and clinical significance for developing targeted therapies and improving long-term graft outcomes. This review aims to systematically explore the molecular mechanisms underlying vein graft failure (VGF) from a pathophysiological perspective, with a particular focus on the role of VSMCs in IH and their potential as therapeutic targets.

## 2 Differences between venous and arterial grafts

There are notable structural and functional differences between venous and arterial grafts, particularly between the GSV and arterial conduits. Histological studies have shown that the medial layer of the IMA contains a significantly higher proportion of elastic fibers compared to the GSV. This structural feature confers greater elasticity and higher compliance to the IMA under arterial pressure (13). Beyond providing mechanical resilience, elastic fibers serve as a physical barrier to VSMCs migration and proliferation toward the intima, thereby mitigating intimal hyperplasia (14). At the site of arteriovenous anastomosis, differences in compliance between the two vessel types can induce hemodynamic disturbances, further exacerbating IH in vein grafts (15). From the perspective of ECM composition, the IMA is rich in heparan sulfate, whereas the GSV predominantly contains chondroitin sulfate. Chondroitin sulfate has a higher affinity for low-density lipoprotein (LDL) and very low-density lipoprotein (VLDL), which may contribute to the increased atherogenicity observed in vein grafts (16, 17). In addition, the GSV exhibits a diminished capacity to secrete vasodilatory substances such as nitric oxide (NO), and its VSMCs are less responsive to these mediators. Collectively, these characteristics increase the susceptibility of vein grafts to stenosis (18).

Biological differences in VSMCs between veins and arteries are also significant. *In vitro* studies have demonstrated that venous VSMCs are smaller, spindle-shaped, and exhibit enhanced proliferative and migratory potential. These cells also display downregulation of α-SMA and upregulation of matrix metalloproteinase-2/9(MMP-2/9), indicating a greater propensity for phenotypic switching toward a synthetic state (19).

To link the structural and biological distinctions of vascular conduits with their clinical implications, it is essential to recognize that commonly used grafts exhibit characteristic performance profiles primarily determined by their intrinsic histological properties. Arterial conduits, such as the LIMA and RA, possess a well-developed elastic lamina, preserved endothelial function, and greater resistance to vasomotor dysfunction, all of which contribute to their superior durability under arterial hemodynamic load. In contrast, venous conduits such as the GSV lack these protective features and are therefore more susceptible to intimal hyperplasia and accelerated atherosclerotic degeneration after arterial implantation (20). These inherent differences also influence practical aspects of conduit selection, including availability, harvesting complexity, and technical considerations during anastomosis. To provide a clear and concise overview of these clinically relevant characteristics, we summarize the major attributes of commonly used grafts—including durability, availability, technical demands, primary indications, and key determinants of graft failure—in Table 1 (21–24).

**Table 1:** Characteristics of commonly used conduits for CABG.

Conduit	Durability/Patency	Availability	Technical difficulty	Primary use	Factors related to failure
LIMA	Highest long-term patency (>90% at 10 years)	Single vessel, limited availability	Moderate; requires careful harvesting	Preferred for LAD	Competitive flow, size mismatch, spasm, distal anastomotic stenosis
RA	Good patency (approximately 90% at 10 years)	Generally available unless prior cannulation	Higher technical difficulty; prone to spasm	Alternative arterial conduit	Vasospasm, inadequate distal runoff, competitive flow, harvesting trauma
GSV	Lowest patency (50%–60% at 10 years)	Abundant and easily accessible	Easy; minimal technical challenge	Most commonly used venous conduit	Endothelial injury, IH, atherosclerosis, vein-wall mismatch, surgical trauma, storage solutions

LIMA, left internal mammary artery; RA, radial artery; GSV, great saphenous vein; LAD, left anterior descending coronary artery; IH, intimal hyperplasia.

**Table 2:** Epigenetic and Non-coding RNAs-mediated regulation of VSMCs phenotypic switching.

Non-coding RNAs/epigenetic factor	Study (Ref No.)	Target/molecular axis	Mechanistic summary	Effect on VSMCs/IH
miR-375-3p/METTL3 (m^6^A modification)	(73)	METTL3 → m^6^A of pri-miR-375 → PDK1	METTL3 knockdown reduces m^6^A on pri-miR-375, suppressing maturation of miR-375-3p; reduced miR-375-3p increases PDK1 expression	Inhibits synthetic phenotype transition; suppresses proliferation/migration
miR-199a-5p	(74)	MMP2	Reduced exosomal miR-199a-5p upregulates MMP2, enhancing synthetic phenotype features	Promotes proliferation, migration, apoptosis-resistance; contributes to vascular remodeling
miR-184-3p	(75)	Cyp26b1	Elevated miR-184-3p degrades Cyp26b1 mRNA, disrupting contractile transcriptome	Promotes proliferation/migration; drives synthetic phenotype
lncRNA ITGA2 (enhancer-associated)	(76)	ITGA2; NONO → H3K27ac	ITGA2-lncRNA recruits NONO, increases H3K27ac at ITGA2 promoter, enhancing enhancer-promoter looping	Strengthens PDGF-BB–induced proliferation/migration; promotes IH
lncRNA VELRP	(77)	WDR5 → H3K4me3 → CDK1/2/4	Binds WDR5 to enhance H3K4me3, activates CDK signaling	Drives contractile → proliferative transition; promotes pathological remodeling
lncRNA H19	(78)	miR-125a-3p → FLT1	H19 sponges miR-125a-3p, relieving repression of FLT1	Promotes synthetic phenotype; increases VSMCs migration + IH
lncDACH1	(79)	HSP90–SRPK1 → AKT signaling	Blocks HSP90 binding to SRPK1, suppressing SRPK1 nuclear translocation and AKT phosphorylation	Inhibits phenotypic switching and IH
circ_0000006	(80)	miR-483-5p → KDM2B	Sponges miR-483-5p, releasing inhibition on KDM2B	Promotes synthetic phenotype
circSETD2/p-414aa peptide	(81)	HuR → C-FOS	circSETD2 encodes peptide p-414aa, which binds HuR and destabilizes C-FOS mRNA	Suppresses phenotypic switching and IH
circSMAD3	(82)	hnRNPA1 → WDR76 → p53γ	circSMAD3 enhances hnRNPA1 ubiquitination; activates p53γ pathway	Inhibits proliferation & synthetic transformation; reduces IH

CDK, cyclin-dependent kinase; C-FOS, C-Fos proto-oncogene; circRNAs, circular RNAs; Cyp26b1, cytochrome P450 family 26 subfamily B member 1; FLT1, fms-like tyrosine kinase 1; H3K27ac, histone H3 lysine 27 acetylation; H3K4me3, histone H3 lysine 4 trimethylation; HSP90, heat shock protein 90; hnRNPA1, heterogeneous nuclear ribonucleoprotein A1; HuR, human antigen R; IH, intimal hyperplasia; ITGA2, integrin alpha-2; KDM2B, lysine demethylase 2B; lncRNAs, long non-coding RNAs; m^6^A, N^6^-methyladenosine; METTL3, methyltransferase-like 3; miRNAs, microRNAs; MMP2, matrix metalloproteinase 2; NONO, non-POU domain-containing octamer-binding protein; PDGF-BB, platelet-derived growth factor-BB; PDK1, phosphoinositide-dependent protein kinase-1; SO_2_, sulfur dioxide; SRPK1, serine/arginine-rich splicing factor protein kinase 1; TAK, Takayasu's arteritis; VELRP, vessel-enriched lncRNA regulated by PDGF-BB; VSMCs, vascular smooth muscle cells; WDR5, WD repeat domain 5; WDR76, WD repeat domain 76.

## 3 Pathological mechanisms of VGF

### 3.1 Early thrombosis

VGF is a complex and dynamic pathological process that evolves over time, with distinct mechanisms predominating at different postoperative stages. In the acute phase (within the first month post-surgery), thrombosis is the primary cause of early saphenous vein graft (SVG) failure, accounting for approximately 10% of cases. This process is closely associated with vascular injury induced by surgical manipulation, including mechanical trauma during vessel harvesting and ischemia–reperfusion injury. Such insults compromise endothelial integrity, expose subendothelial collagen, and activate the extrinsic coagulation cascade. Concurrently, the secretion of vasodilators such as NO and prostacyclin (PGI_2_) is reduced, while levels of procoagulant and vasoconstrictive factors such as endothelin-1 and thromboxane A2 increase, disrupting the delicate balance between coagulation and anticoagulation (25). Additionally, hemodynamic abnormalities due to technical errors in anastomosis or size mismatch between the graft and target vessel further elevate the risk of thrombosis (6). Clinically, early graft thrombosis typically presents as perioperative myocardial infarction, hemodynamic instability, or acute graft occlusion identified on urgent angiography, and often necessitates immediate reintervention (26).

### 3.2 Intimal hyperplasia

During the intermediate phase (1 month to 1 year postoperatively), IH becomes the predominant mechanism of graft failure. IH represents an abnormal adaptive response of the vein to the high-pressure arterial environment and is characterized by the phenotypic switch of VSMCs from a quiescent, contractile state to an active, synthetic phenotype (21). Following endothelial injury, the expression of cytokines such as platelet-derived growth factor (PDGF) and transforming growth factor-β (TGF-β), along with inflammatory mediators like interleukin-6 (IL-6), is upregulated, promoting VSMCs migration and proliferation within the intimal layer. Simultaneously, excessive deposition of ECM components—including collagen and elastin—contributes to progressive IH and luminal narrowing (27, 28). This process is particularly pronounced at anastomotic sites and may be driven by alterations in local shear stress (28). In clinical practice, IH-related stenosis typically manifests as recurrent angina or inducible ischemia on noninvasive stress testing and is usually confirmed by CT angiography or invasive coronary angiography. Patient-related factors such as diabetes, dyslipidemia, small graft diameter, and smoking further increase the likelihood of intermediate graft failure (29).

### 3.3 Accelerated atherosclerosis

**Figure 1:**
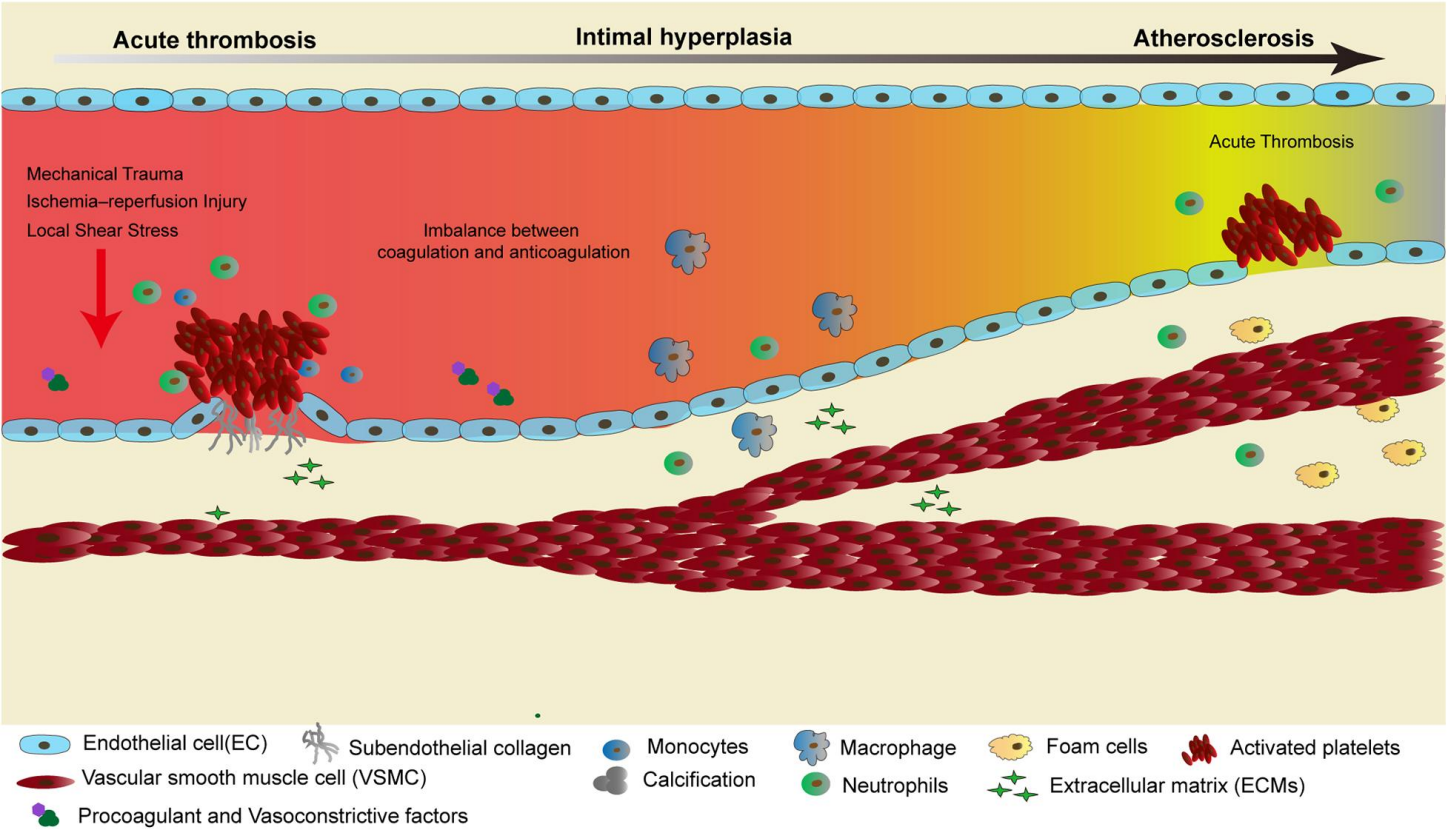
The evolution of vein graft failure: a cascade from early thrombosis to late atherosclerotic rupture. After graft implantation, mechanical injury, ischemia–reperfusion, and disturbed shear stress compromise endothelial integrity and expose subendothelial collagen, initiating platelet activation and early thrombosis. As inflammatory signaling intensifies, VSMCs undergo phenotypic switching, migrate and proliferate, and deposit extracellular matrix, driving progressive intimal hyperplasia. In the late postoperative period, the chronically inflamed and remodeled neointima promotes accelerated atherosclerosis and plaque instability, ultimately predisposing the graft to rupture-related failure.

In the late postoperative phase (beyond 1 year), accelerated atherosclerosis becomes the leading cause of SVG failure, progressing at a significantly faster rate than in native coronary arteries. The pro-atherogenic microenvironment established by preexisting IH facilitates this progression. Compared with native vessel lesions, atherosclerotic plaques in SVGs exhibit distinct pathological features: they are diffusely and concentrically distributed, rich in foam and inflammatory cells, possess thin fibrous caps, and display minimal calcification (7) (Figure 1). Clinically, late graft failure typically manifests as recurrent ischemia or acute coronary syndromes, largely attributable to the fragile and rupture-prone nature of SVG atherosclerotic plaques (30). Long-term graft patency therefore depends not only on favorable biological remodeling but also on optimized medical therapy, including intensive lipid-lowering, antiplatelet therapy, and strict control of cardiovascular risk factors.

### 3.4 Factors regulating graft maturation and failure

Graft maturation refers to the adaptive process by which a venous conduit acquires arterial characteristics, restoring endothelial integrity, achieving hemodynamic stability, and developing the structural resilience required for long-term patency. This process is regulated by the dynamic balance between vascular repair responses and the biomechanical forces encountered after arterial implantation. Several key determinants collectively dictate whether the graft undergoes adaptive remodeling or progresses toward failure. Endothelial recovery is fundamental to early graft healing. Rapid re-endothelialization suppresses platelet activation and inflammation, whereas delayed endothelial repair—caused by impaired eNOS activity or inadequate endothelial progenitor cell recruitment—predisposes the graft to early thrombosis (13, 15). Hemodynamic adaptation is crucial for long-term graft stability. Proper arterialization of the venous wall promotes physiological remodeling, while disturbed or oscillatory shear stress at anastomotic sites triggers aberrant VSMCs activation and drives IH (31). ECM remodeling further shapes graft architecture and biomechanical compliance. Dysregulation of MMPs activity may either exacerbate ECM deposition and luminal narrowing or enhance matrix degradation, facilitating VSMCs migration. Inflammatory and immune responses strongly influence the healing microenvironment (32). Pro-inflammatory macrophage polarization drives VSMCs phenotypic switching and neointimal formation, whereas reparative immune profiles favor constructive remodeling (33). Surgical and technical factors, including conduit handling, preservation strategies, ischemia duration, and anastomotic mismatch, largely determine the extent of initial endothelial injury and significantly shape subsequent graft outcomes (7). Together, these factors constitute the regulatory framework that determines whether a graft matures successfully or progresses toward thrombosis, progressive IH, and late atherosclerotic degeneration.

## 4 The role of VSMCs phenotypic switching in IH of vein grafts

VSMCs, the primary structural components of the tunica media, are essential for maintaining vascular homeostasis. Under physiological conditions, VSMCs display a contractile phenotype, characterized by an elongated spindle-shaped morphology and high expression of contractile proteins such as α-SMA and SM22α. This differentiated phenotype enables VSMCs to regulate vascular tone while maintaining low proliferative and migratory activity (34). However, when vein grafts are exposed to the arterial circulation, VSMCs undergo a phenotypic switch in response to various pathological stimuli. These stimuli include elevated arterial pressure, increased shear stress, and exposure to a pro-inflammatory and growth factor-rich microenvironment, comprising factors such as PDGF, tumor necrosis factor-α (TNF-α), and IL-6 (9). Under such conditions, VSMCs progressively lose their contractile features, evidenced by downregulation of contractile protein expression and a morphological shift from spindle-shaped to irregular polygonal forms. Concurrently, genes associated with the synthetic phenotype—including MMPs and osteopontin (OPN)—are markedly upregulated (35). This transformation enhances the migratory and proliferative capacity of VSMCs, facilitating their movement into the intimal layer through degradation of the ECM, followed by abnormal proliferation beneath the endothelium. Additionally, synthetic VSMCs acquire an active secretory profile, producing large quantities of ECM proteins and pro-inflammatory mediators. Together, these processes contribute to the onset and progression of IH in vein grafts (18). While the classical contractile-to-synthetic transition remains the primary phenotypic axis driving IH, recent lineage-tracing and single-cell transcriptomic studies have shown that VSMCs can adopt multiple intermediate or alternative states in response to arterialization and injury, including macrophage-like, foam cell, mesenchymal stem-like, myofibroblast-like, and osteochondral-like phenotypes (36). Although the functional significance of these subpopulations in vein graft remodeling is not yet fully defined, emerging evidence underscores the remarkable phenotypic plasticity of VSMCs and suggests that distinct VSMCs-derived states may differentially contribute to IH progression.

Despite these advances, the regulatory mechanisms governing VSMCs phenotypic switching remain incompletely understood. Elucidating these molecular pathways holds substantial scientific and clinical value for developing targeted strategies to prevent or treat IH in vein grafts following CABG, ultimately improving long-term graft patency.

## 5 Regulatory mechanisms of VSMCs phenotypic switching

### 5.1 Role of endothelial cells (ECs) dysfunction

IH, a critical pathological process leading to VGF, is initiated by ECs dysfunction. During vein harvesting and graft implantation, surgical trauma, ischemia–reperfusion injury, and vasospasm contribute to ECs activation. Upon exposure to the arterial environment, this activation is further exacerbated. Activated ECs upregulate the expression of cell adhesion molecules (e.g., VCAM-1, ICAM-1) and chemokines (e.g., MCP-1), thereby promoting the recruitment and infiltration of immune cells such as monocytes and lymphocytes—events that trigger IH and vascular remodeling (13, 37, 38).

ECs and VSMCs are functionally coupled through both direct intercellular contact and paracrine signaling, cooperatively regulating vascular tone, cell proliferation, and inflammatory responses. Under physiological conditions, NO released from ECs helps maintain the contractile phenotype of VSMCs and inhibits their transition to the synthetic phenotype (39). However, ECs injury impairs the activity of endothelial nitric oxide synthase (eNOS) and cystathionine γ-lyase (CSE), leading to reduced production of NO and hydrogen sulfide (H_2_S). The depletion of these gasotransmitters not only enhances inflammatory cell infiltration but also facilitates VSMCs phenotypic switching, thereby accelerating the progression of IH (38, 40). Furthermore, in the inflammatory microenvironment established by activated ECs, platelets become activated and secrete pro-inflammatory mediators such as IL-6, IL-8, thromboxane A2 (TXA2), and TNF-α, along with growth factors like PDGF and fibroblast growth factor (FGF). These factors collectively drive aberrant VSMCs proliferation and migration (28, 41, 42). In addition, ECs and VSMCs interact indirectly via secretion of ECM proteins, including collagen and fibronectin, which further facilitate VSMCs phenotypic modulation (25, 43). A positive feedback loop amplifies the local inflammatory response: initial ECs dysfunction induces the release of pro-inflammatory cytokines, which in turn exacerbate ECs impairment—forming a vicious cycle of “ECs dysfunction → inflammatory infiltration → VSMCs activation” (37, 44).

In summary, the pathological cascade of IH begins with ECs activation, followed by VSMCs migration and proliferation into the intimal layer and ECM remodeling. Therefore, strategies aimed at restoring ECs function, enhancing the secretion of anti-intimal hyperplasia factors, or inhibiting ECs-mediated pro-intimal hyperplasia signaling pathways hold promise as effective approaches for the prevention and treatment of IH in vein grafts.

### 5.2 PDGF-BB

PDGF-BB, a key member of the PDGF family, is a peptide growth factor with diverse biological functions. Under physiological conditions, its expression in the vascular system remains relatively low and is primarily synthesized by VSMCs, fibroblasts, and macrophages (45). However, in response to vascular injury or pathological stimuli—such as inflammation or oxidative stress—PDGF-BB expression increases markedly, becoming a central regulator of VSMCs proliferation, migration, and phenotypic switching (46).

PDGF-BB facilitates the transition of VSMCs from a contractile to a synthetic phenotype through multiple molecular mechanisms. Experimental studies have demonstrated that PDGF-BB stimulation significantly enhances VSMCs migratory and proliferative capacities while promoting abnormal ECM accumulation. At the molecular level, this process is associated with upregulation of cyclin D1 and cyclin-dependent kinase 4 (CDK4), along with downregulation of the cyclin-dependent kinase inhibitor p27. Pretreatment with a p90 ribosomal S6 kinase (p90RSK) inhibitor effectively blocks these effects, highlighting the essential role of p90RSK in PDGF-BB-mediated cell cycle regulation (47). Further mechanistic insights were provided by Huang et al., who identified the specific signaling pathway through which PDGF-BB regulates VSMCs function. Their study demonstrated that PDGF-BB markedly upregulates inositol 1,4,5-trisphosphate receptor type 3 (IP3R3) and promotes phosphorylation of cAMP response element-binding protein via activation of calcium/calmodulin-dependent protein kinase II and AKT, ultimately modulating VSMCs proliferation, phenotypic switching, and IH (48). Clinical investigations have also revealed a significant positive correlation between PDGF-BB expression and the severity of IH in vein grafts (49).

PDGF-BB, recognized as the most potent mitogen for VSMCs, is widely used to model phenotypic switching in experimental studies. A thorough understanding of PDGF-BB–mediated signaling pathways may provide essential theoretical support for the development of targeted therapies to prevent VGF.

### 5.3 Signaling pathways

**Figure 2:**
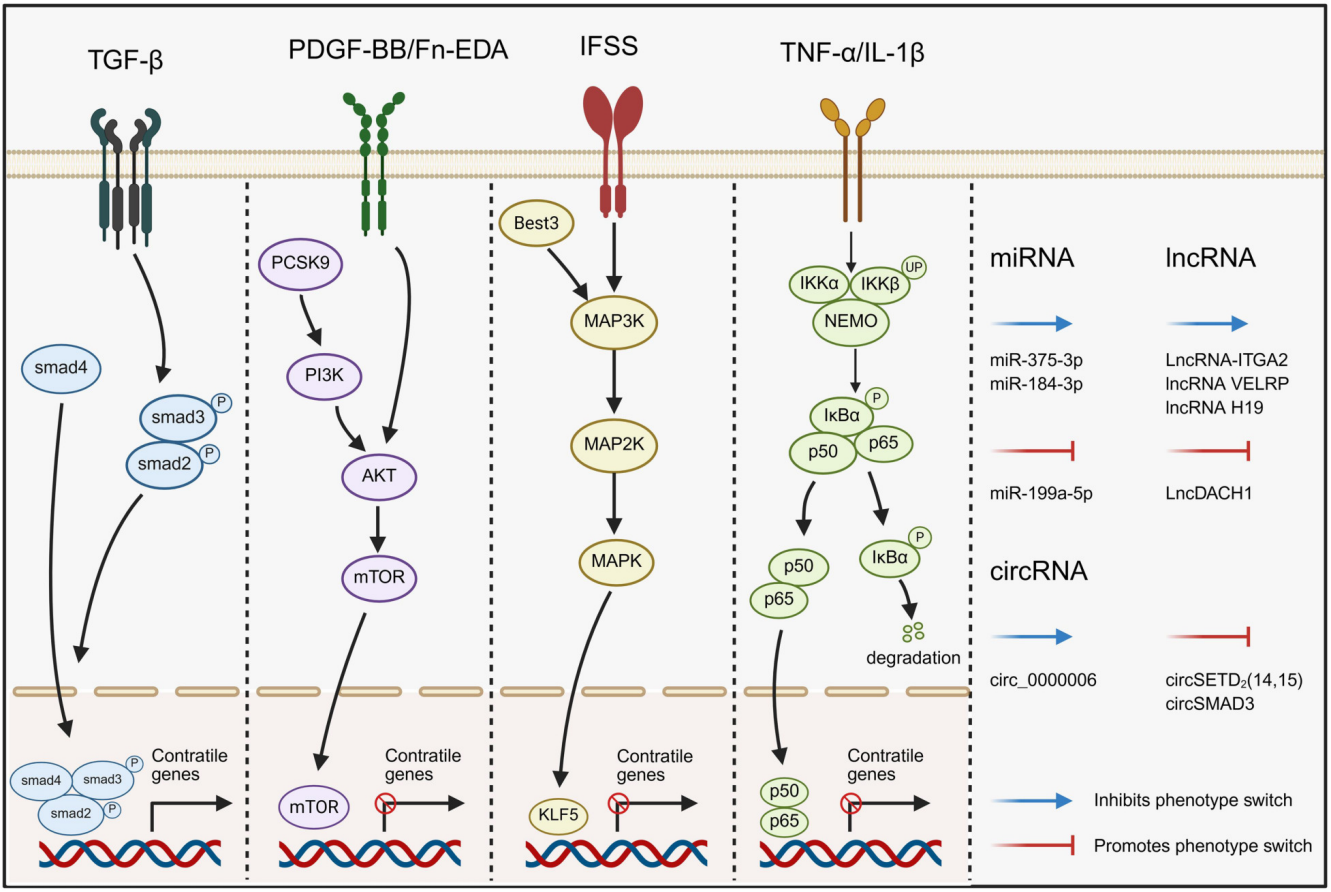
Mechanisms underlying VSMCs phenotypic switching. The mechanism is initiated by endothelial dysfunction and amplified by the key driver PDGF-BB. It involves the integration of multiple signaling pathways (MAPK, mTOR, NF-κB, TGF-β) and a complex network of non-coding RNAs (miRNAs, lncRNAs, circRNAs), which collectively orchestrate the transition from a contractile to a synthetic phenotype. Created in BioRender. Store, B. (2026) https://BioRender.com/1vm11ig, licensed under Academic License.

The phenotypic switching of VSMCs is profoundly influenced by multiple signal transduction pathways (Figure 2), including TGF-β, mitogen-activated protein kinase (MAPK), mammalian target of rapamycin (mTOR), and Nuclear factor-kappa B (NF-κB).

#### 5.3.1 TGF-β signaling pathway

TGF-β is a critical signaling molecule involved in numerous biological processes, including cell proliferation, differentiation, immune regulation, and inflammatory responses (50). The canonical TGF-β signaling pathway is initiated by the binding of TGF-β to the type II receptor (TβRII) on the cell membrane. This interaction leads to the recruitment and phosphorylation of the type I receptor (TβRI), which subsequently phosphorylates the intracellular mediators Smad2 and Smad3. The phosphorylated Smad2/3 proteins then form a transcriptional complex with Smad4, which translocates into the nucleus to regulate the expression of target genes, such as those encoding contractile proteins in VSMCs (51). This signaling cascade plays a crucial role in preserving the contractile phenotype of VSMCs (52). Experimental studies have demonstrated that smooth muscle cell-specific deletion of TGF-β receptors results in VSMCs dedifferentiation, promotes neointimal formation, and contributes to the development of aortic aneurysms (53). Furthermore, during vascular remodeling, the expression of tenascin-X (TN-X) in VSMCs is significantly upregulated. In VSMCs-specific TN-X knockout mice, enhanced TGF-β signaling activity was observed, accompanied by increased expression of VSMCs differentiation markers and reduced neointima formation. Notably, this phenotype could be reversed by anti-TGF-β antibody treatment or deletion of TGF-β receptors (54).

These findings underscore the central role of TGF-β signaling in maintaining VSMCs differentiation and provide insights into potential therapeutic targets for vascular remodeling-related diseases.

#### 5.3.2 MAPK signaling pathway

The MAPK signaling pathway plays a central regulatory role in the phenotypic switching of VSMCs. This pathway primarily involves members of the serine/threonine protein kinase family, including extracellular signal-regulated kinase 1/2 (ERK1/2), c-Jun N-terminal kinase (JNK), and p38 MAPK. These kinases transduce extracellular stimuli into intracellular signals and regulate key biological processes such as VSMCs proliferation, differentiation, and inflammatory responses (55). Studies have shown that inhibition of MAPK pathway activation effectively preserves the contractile phenotype of VSMCs. In particular, activation of p38 MAPK is a critical molecular event in PDGF-induced transformation from the contractile to the synthetic phenotype (56). Moreover, deletion of Bestrophin 3 (Best3) leads to increased phosphorylation of MEKK2/3, which activates the downstream MAPK cascade and significantly reduces the fibromyocyte-like VSMCs subpopulation. Restoration of Best3 expression or inhibition of MEKK2/3 effectively suppresses phenotypic switching (57). Gao et al. combined numerical simulations and *in vitro* experiments to demonstrate that interstitial flow shear stress (IFSS) activates the epidermal growth factor receptor (EGFR), which subsequently triggers the MAPK pathway, promotes nuclear translocation of the transcription factor Kruppel-like factor 5 (KLF5), and ultimately drives phenotypic switching and secretion of pro-calcification extracellular vesicles (EVs). Pharmacological inhibition of the MAPK pathway blocks KLF5 nuclear accumulation and helps maintain the contractile phenotype of VSMCs (58).

Taken together, these findings underscore the pivotal role of the MAPK signaling pathway in VSMCs phenotypic switching and suggest promising therapeutic targets for vascular remodeling. Targeted modulation of this pathway may offer a novel strategy to preserve the contractile phenotype and prevent pathological phenotypic switching.

#### 5.3.3 mTOR signaling pathway

The mTOR is a serine/threonine kinase that functions as a central hub for intracellular signal transduction. It integrates various extracellular stimuli—including nutrients, growth factors, and energy status—to regulate critical cellular processes such as growth, proliferation, metabolism, and survival (59). mTOR primarily forms two distinct functional complexes: mTOR complex 1 (mTORC1) and mTOR complex 2 (mTORC2). mTORC1 modulates protein synthesis by phosphorylating downstream targets such as ribosomal protein S6 kinase (S6K) and eukaryotic translation initiation factor 4E-binding protein 1 (4E-BP1), thereby promoting cell growth and proliferation. In contrast, mTORC2 is primarily involved in cell survival, cytoskeletal remodeling, and migration (60). In VSMCs, the precise regulation of mTOR signaling is essential for maintaining phenotypic stability. Dysregulation—whether through hyperactivation or suppression—can induce VSMCs phenotypic switching. Studies have shown that stimulation with PDGF-BB markedly increases mTOR phosphorylation in VSMCs. This is accompanied by upregulation of proliferation-associated markers such as cyclin D1 and proliferating cell nuclear antigen (PCNA), as well as migration-related proteins including MMP2/9. Simultaneously, contractile markers such as α-SMA and SM22α are downregulated. In carotid artery balloon injury models, this phenotypic switch contributes to pronounced neointimal hyperplasia, which can be effectively attenuated by mTOR pathway inhibitors such as calcitonin (CT) (61). Moreover, proprotein convertase subtilisin/kexin type 9 (PCSK9) has been shown to activate the phosphatidylinositol 3-kinase/protein kinase B/mTOR (PI3K/AKT/mTOR) pathway, thereby promoting VSMCs phenotypic switching and IH (62). Jain et al. further demonstrated that fibronectin extra domain A (Fn-EDA), derived from VSMCs, can activate the AKT1/mTOR pathway through a synergistic mechanism involving integrins and Toll-like receptor 4 (TLR4), thereby accelerating the switch from the contractile to the synthetic phenotype and contributing to neointimal lesion formation (63).

In summary, the mTOR signaling pathway plays a pivotal role in the regulation of VSMCs phenotypic switching. Elucidating its molecular mechanisms not only enhances our understanding of vascular remodeling but also provides a promising foundation for the development of targeted therapeutic strategies aimed at preventing vascular graft failure and related pathologies.

#### 5.3.4 NF-κB signaling pathway

NF-κB is a highly conserved family of transcription factors in eukaryotes, comprising subunits such as RelA (p65), RelB, c-Rel, p50, and p52. It plays a pivotal role in regulating immune responses, inflammation, and the balance between cell proliferation and apoptosis (64). Under resting conditions, the NF-κB dimer—typically composed of p50 and p65—is sequestered in the cytoplasm by binding to the inhibitory protein IκB (inhibitor of kappa B), thereby maintaining its inactive state. Upon stimulation by TNF-α, IL-1β, or pathogen-associated molecular patterns (PAMPs), the IκB kinase (IKK) complex becomes activated, leading to the phosphorylation, ubiquitination, and subsequent degradation of IκB. This process facilitates the nuclear translocation of the NF-κB dimer, where it binds to promoter regions of target genes encoding pro-inflammatory cytokines, adhesion molecules, and anti-apoptotic proteins, thereby initiating transcriptional programs (65). NF-κB activation contributes to VSMCs proliferation and migration, enhances inflammatory cell infiltration, and promotes a vicious cycle that accelerates vascular remodeling (38). Numerous studies have demonstrated the critical role of NF-κB signaling in the phenotypic switching of VSMCs. For instance, exposure to tetrachlorobisphenol A (TCBPA) activates NF-κB, induces abnormal VSMCs proliferation, and amplifies inflammatory cascades (66). Furthermore, NF-κB activation promotes the phenotypic shift of VSMCs from a contractile to a synthetic state, and cooperates with the inflammatory microenvironment to drive disease progression (67). Mao et al. reported that exosomes derived from quercetin-conjugated pancreatic stellate cells (PSCs) inhibit carotid IH after balloon injury in rats by modulating NF-κB signaling (68). Bai et al. found that the circular RNA circACTA2 can bind directly to the p50 subunit, preventing nuclear translocation of the p50/p65 heterodimer. This inhibition suppresses activation of the NOD-like receptor thermal protein domain associated protein 3 (NLRP3) inflammasome and reduces the secretion of pro-inflammatory cytokines such as IL-1β and IL-18. Consequently, this mechanism prevents gasdermin D (GSDMD)-mediated pyroptosis in VSMCs, preserves their contractile phenotype, and reduces IH following vascular injury (69). Moreover, inflachromene (ICM), an inhibitor of high mobility group box proteins 1 and 2 (HMGB1/2), attenuates Ang II-induced nucleocytoplasmic translocation of HMGB1/2, downregulates TLR4 expression and NF-κB phosphorylation, thereby inhibiting VSMCs phenotypic switching (70).

In summary, the NF-κB signaling pathway plays a central role in regulating inflammation and VSMCs phenotypic modulation during vascular remodeling. A variety of stimuli can activate this pathway in VSMCs, promoting their transition to a synthetic phenotype and accelerating pathological remodeling. Targeting key molecules within the NF-κB pathway—such as circACTA2 and HMGB1/2—may help preserve the contractile phenotype of VSMCs and offers promising therapeutic strategies for vascular remodeling–related diseases.

### 5.4 Regulatory role of Non-coding RNAs

Non-coding RNAs, important epigenetic regulators, primarily include microRNAs (miRNAs), long non-coding RNAs (lncRNAs), and circular RNAs (circRNAs). These molecules participate in the regulation of VSMCs phenotypic switching through intricate molecular mechanisms and play essential roles in the onset and progression of cardiovascular diseases (Figure 2).

#### 5.4.1 The role of miRNAs in VSMCs phenotypic switching

MiRNAs are a class of endogenous small non-coding RNAs, typically 19–25 nucleotides in length. They primarily function by binding complementarily to the 3′-untranslated region (3′-UTR) of target mRNAs, thereby inhibiting their translation or promoting their degradation, and ultimately exerting post-transcriptional negative regulation of gene expression (71). Aberrantly expressed miRNAs can influence VSMCs proliferation, migration, inflammation, apoptosis, calcification, and oxidative stress by targeting key genes involved in maintaining either the contractile or synthetic phenotype, and thus play pivotal roles in vascular remodeling and cardiovascular pathology (72). For instance, silencing methyltransferase-like 3 (METTL3) reduces the N^6^-methyladenosine (m^6^A) modification of pri-miR-375, weakens DiGeorge syndrome critical region 8 (DGCR8) binding, and inhibits the maturation of miR-375-3p. As miR-375-3p negatively regulates phosphoinositide-dependent protein kinase-1 (PDK1), its downregulation leads to increased PDK1 expression, ultimately suppressing the transition of VSMCs from a contractile to a synthetic phenotype (73). Similarly, Guo et al. demonstrated that serum exosomal miR-199a-5p levels were significantly reduced in patients with Takayasu's arteritis (TAK), resulting in the upregulation of its target gene, MMP2. This upregulation promotes the phenotypic switch of VSMCs toward the synthetic state, thereby enhancing cell migration, proliferation, and resistance to apoptosis, which collectively contribute to inflammatory infiltration and structural remodeling of the vascular wall (74). Additionally, miR-184-3p functions as a crucial effector in the endogenous sulfur dioxide (SO_2_) signaling pathway. Under conditions of SO_2_ pathway inhibition, miR-184-3p expression is markedly elevated. By targeting and degrading cytochrome P450 family 26 subfamily B member 1 (Cyp26b1) mRNA, it disrupts transcriptional programs essential for maintaining the contractile phenotype, promoting VSMCs proliferation, migration, and phenotypic switching. Importantly, treatment with miR-184-3p inhibitors restores Cyp26b1 expression and reverses VSMCs dysfunction caused by aberrant SO_2_ signaling (75).

#### 5.4.2 The role of lncRNA in VSMCs phenotypic switching

LncRNA can participate in the molecular mechanism of VSMCs phenotypic switching through multiple ways such as epigenetic modification, competing endogenous RNA mechanism and signal pathway regulation, and plays a vital role in regulating the behaviors of VSMCs. Studies have shown that enhancer-associated lncRNA-ITGA2 (lncRNA-integrin alpha-2) is significantly upregulated after vascular injury. It interacts with the non-pou domain containing octamer-binding protein (NONO), promotes the histone H3 lysine 27 acetylation (H3K27ac) modification in the promoter region of ITGA2, and strengthens the interaction between the enhancer and the promoter, thus activating the transcriptional expression of ITGA2 (76). This regulatory process not only amplifies the proliferation and migration signals of VSMCs induced by PDGF-BB, but also significantly promotes the phenotypic switching of VSMCs from the contractile phenotype to the synthetic phenotype, ultimately leading to excessive IH and vascular pathological remodeling. Another study identified the vessel-enriched lncRNAs regulated by PDGF-BB (VELRP), which can bind to WD repeat domain 5 (WDR5) to promote the trimethylation of H3K4 modification, activate the CDK 1/2/4 signaling pathway, and thus drive the transformation of pulmonary artery smooth muscle cells from the contractile phenotype to the proliferative phenotype, inducing pulmonary vascular pathological remodeling (77). Jiang et al. found that lncRNA H19 is significantly upregulated in damaged vascular tissues. It competitively binds to miR-125a-3p, relieves the inhibitory effect of the latter on fms-like tyrosine kinase 1 (FLT1), promotes the transformation of VSMCs to the synthetic phenotype, and then enhances the proliferation and migration abilities of cells, exacerbating IH (78). In addition, LncDACH1 can competitively inhibit the binding of heat shock protein 90 (HSP90) to serine/arginine-rich splicing factor protein kinase 1 (SRPK1) block the nuclear translocation of SRPK1 and the phosphorylation process of AKT, thereby inhibiting the phenotypic switching of VSMCs and IH (79).

#### 5.4.3 The role of circRNA in VSMCs phenotypic switching

CircRNAs are a unique class of non-coding RNAs characterized by their covalently closed circular structure, which confers high stability and tissue-specific expression patterns. Recent studies have demonstrated that the expression of circ_0000006 is significantly upregulated during vascular remodeling. It functions by sponging miR-483-5p, thereby relieving the inhibition of lysine demethylase 2B (KDM2B) and promoting the phenotypic switch of VSMCs from a contractile to a synthetic state (80). Wang et al. further confirmed that circSETD_2_ (14, 15) can encode a novel protein, p-414aa. This protein binds to human antigen R (HuR) and destabilizes the mRNA of the C-Fos proto-oncogene (C-FOS), thereby significantly suppressing the contractile-to-synthetic phenotypic switching of VSMCs and inhibiting abnormal cell proliferation and neointimal formation (81). In addition, circSMAD3, derived from the SMAD3 gene, is markedly downregulated in vascular injury models. It interacts with heterogeneous nuclear ribonucleoprotein A1 (hnRNPA1) and promotes its ubiquitination and degradation mediated by WDR76. This, in turn, facilitates the splicing of the precursor RNA of tumor protein p53 and activates the p53γ signaling pathway, ultimately inhibiting the aberrant proliferation and phenotypic switching of VSMCs and reducing neointimal formation *in vivo* (82).

To facilitate comparison and provide an integrated overview of the evidence discussed above, representative studies describing non-coding RNAs-mediated regulation of VSMCs phenotypic switching, including molecular targets and functional consequences, are summarized in Table 2.

In summary, non-coding RNAs participate in the regulation of VSMCs phenotypic switching through diverse molecular mechanisms. Further elucidation of their roles may not only deepen our understanding of IH but also provide theoretical foundations and therapeutic targets for developing effective intervention strategies.

## 6 Treatment of IH

**Figure 3:**
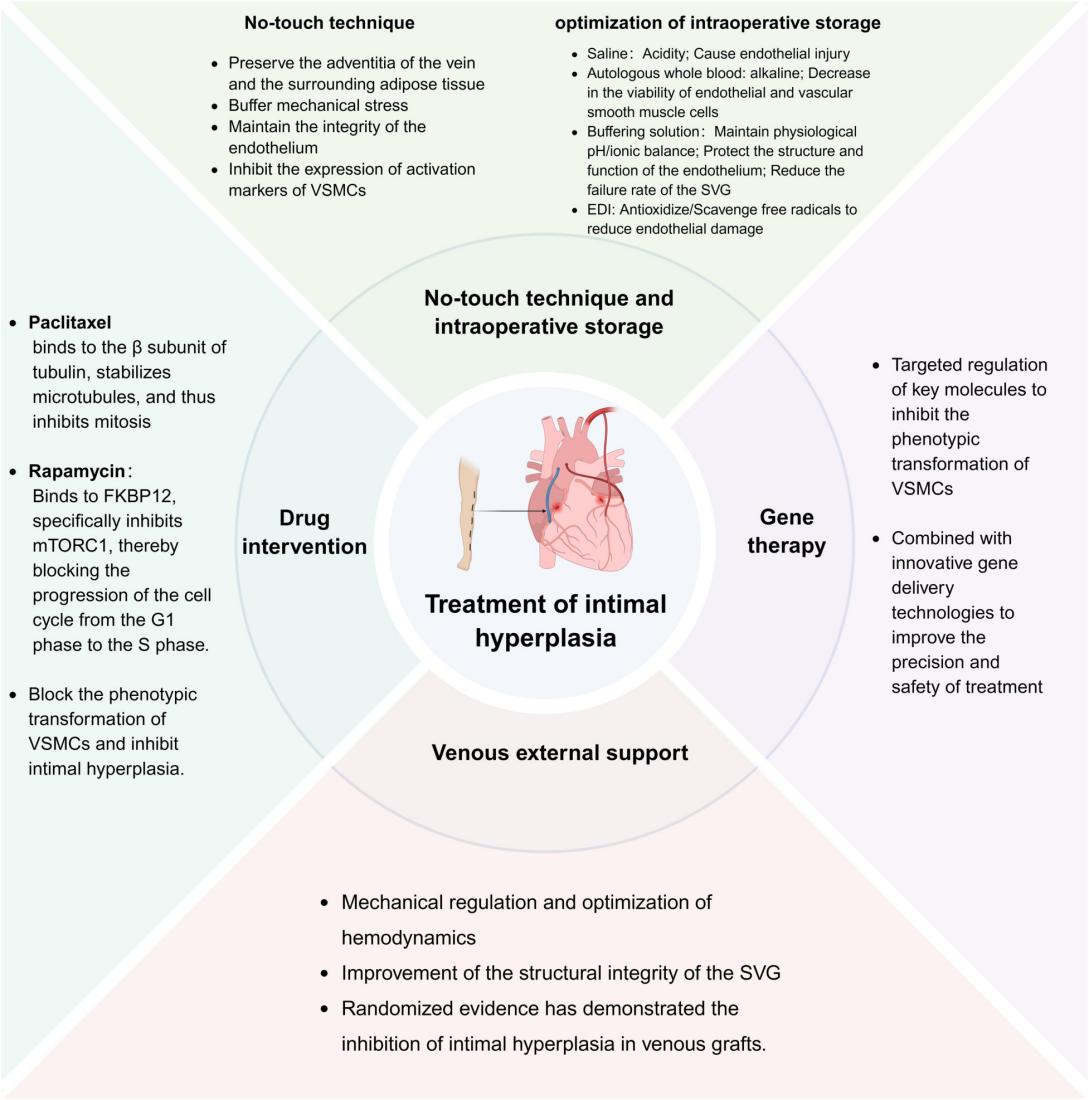
Multimodal therapeutic strategies for IH. The schematic diagram illustrates how no-touch harvesting, optimized storage solutions, pharmacotherapy (e.g., Paclitaxel, Rapamycin), gene therapy, and external stenting inhibit IH by protecting vascular integrity, targeting pathological molecular pathways, regulating VSMCs phenotypic switching, and optimizing hemodynamic environment. These approaches provide a diversified portfolio for improving vein graft patency. Created in BioRender. Store, B. (2026) https://BioRender.com/il3mx21, licensed under Academic License.

IH in vein grafts is the primary pathological process responsible for medium- to long-term graft failure following CABG. Recently, strategies for its prevention and treatment have progressively shifted from traditional, passive interventions toward a comprehensive, multistage, and multitargeted approach to active management. This modern paradigm aims to provide continuous protection throughout the entire graft lifecycle, from surgical harvesting to postoperative remodeling. The core principles include: (1) preventing initial damage by maximizing the protection of vascular structure and function through the no-touch technique and optimized storage solutions; (2) regulating cellular behavior by precisely inhibiting the abnormal activation, proliferation, and phenotypic switching of VSMCs via pharmacological and gene therapies; (3) optimizing the local environment by improving hemodynamics and providing mechanical support with external vascular stents. These complementary strategies form a synergistic and multifaceted therapeutic framework with considerable promise for significantly improving long-term graft patency rates (Figure 3).

### 6.1 No-touch technique

Conventional methods for harvesting veins typically involve stripping the adventitia and surrounding tissues. During the procedure, considerable mechanical traction and dilation are applied, often resulting in vascular endothelial injury and inflammatory responses. These manipulations compromise the structural and functional integrity of the graft, increase the risk of postoperative thrombosis, and promote late-stage IH, thereby adversely affecting long-term graft patency and patient outcomes (83). In contrast, the no-touch vein harvesting technique preserves the anatomical integrity of the adventitia and surrounding perivascular adipose tissue. This protective envelope buffers the mechanical stress imposed by arterial pressure on the venous wall, thereby maintaining endothelial function and significantly reducing the incidence of vascular injury-related complications (7, 84).

Microscopic examination of histological samples can reveal the morphological and biological bases underlying the clinical differences between these two techniques. On light microscopy, endothelial cushions were observed in no-touch saphenous vein grafts (NT-SVGs), whereas the endothelial surface appeared flattened with disrupted integrity in conventional saphenous vein grafts (CON-SVGs) (85). The proposed protective mechanisms include the preservation of eNOS activity, which is abundantly expressed in the adventitia, and the retention of perivascular adipose tissue markers such as leptin and adiponectin (85, 86). Under transmission electron microscopy, VSMCs in the media of NT-SVGs exhibit normal morphology, whereas those in CON-SVGs display irregular features (87). Furthermore, other studies have reported that CON-SVGs express markers indicative of VSMCs activation, which may act as precursors of IH (88).

These protective mechanisms ultimately yield marked improvements in clinical outcomes. the multicenter randomized controlled PATENCY trial, involving 2,655 patients, systematically evaluated the clinical efficacy of the no-touch technique vs. conventional harvesting in CABG. Follow-up data revealed that venous graft occlusion rates were significantly lower in the no-touch group at both 3 and 12 months postoperatively (3 months: 2.8% vs. 4.8%, OR = 0.57, 95% CI: 0.41–0.80, *P* < 0.001; 12 months: 3.7% vs. 6.5%, OR = 0.56, 95% CI: 0.41–0.76, *P* < 0.001). At 12 months, the recurrence of angina was also significantly reduced (2.3% vs. 4.1%, OR = 0.55, 95% CI: 0.35–0.85, *P* < 0.01) (89). Further follow-up at 3 years confirmed sustained clinical benefits, with a continued reduction in graft occlusion rates (5.7% vs. 9.0%, OR = 0.62, 95% CI: 0.48–0.80, *P* < 0.001) and secondary endpoints such as non-fatal myocardial infarction (1.2% vs. 2.7%, *P* = 0.01), repeat revascularization (1.1% vs. 2.2%, *P* = 0.03), recurrent angina (6.2% vs. 8.4%, *P* = 0.03), and readmission due to cardiac causes (7.1% vs. 10.2%, *P* = 0.004) (90). Although no significant differences were observed in all-cause mortality or stroke incidence, these findings demonstrate that the no-touch technique can significantly improve mid-term clinical outcomes in CABG patients.

### 6.2 Conduit storage

Ischemic injury to the vein grafts during the period from harvesting to anastomosis is a key factor in the development of IH. The optimization of intraoperative storage solutions aims to provide a physiological “dormant” environment for the vessel, thereby alleviating metabolic stress and endothelial dysfunction during the ischemic interval. Currently, vascular storage solutions primarily include heparinized 0.9% normal saline, autologous whole blood (AWB), buffer solutions, and endothelial damage inhibitors (EDIs). In clinical practice, heparinized normal saline or AWB is traditionally used (91). However, normal saline is acidic (pH 5.5) and may induce endothelial damage. Additionally, due to environmental changes, the pH of extracorporeal blood rapidly becomes alkaline, and even short-term exposure to a mildly alkaline environment (pH 8.0) can reduce the viability of endothelial and smooth muscle cells (92). While some studies suggest that AWB is more effective than normal saline in reducing endothelial injury and demonstrates superior functional performance, other studies report no significant difference between the two (83).

Buffer solutions—such as the University of Wisconsin solution, TiProtec, and He solution—can better maintain ion homeostasis and physiological pH. These solutions provide superior protection of endothelial structure and function compared to AWB and normal saline (93). Furthermore, they may reduce the failure rate of great SVGs and improve clinical outcomes (94). EDIs, which are buffer solutions developed based on the GALA formula (containing reduced glutathione, L-ascorbic acid, and L-arginine), exhibit antioxidant properties, scavenge free radicals, and support eNOS activity (95, 96). Recent studies have demonstrated that EDIs outperform standard buffer solutions, normal saline, and AWB in minimizing endothelial damage and reducing reactive oxygen species (ROS) levels. Their use is also associated with the alleviation of hypoxic injury and improved redox potential (97, 98).

Comprehensive evidence indicates that optimizing buffer solutions—particularly the EDI formulation—by maintaining ionic balance, reducing oxidative stress, and preserving endothelial function may represent an effective strategy to improve venous graft outcomes. However, large-scale clinical trials are still required to confirm its long-term benefits.

### 6.3 Drug intervention

Drug interventions directly target the cellular and molecular basis of IH—specifically, the abnormal proliferation and phenotypic switching of VSMCs. Currently, the primary pharmacological agents include microtubule stabilizers (e.g., paclitaxel-based drugs) and mTOR signaling inhibitors (e.g., rapamycin-based drugs).

Paclitaxel-based drugs functions primarily by binding to the β-subunit of tubulin, stabilizing polymerized microtubules, preventing their depolymerization, and ultimately arresting mitosis, thereby suppressing VSMCs proliferation and migration (99, 100). Clinical evidence demonstrates that paclitaxel-coated devices significantly improve outcomes compared to plain old balloon angioplasty or bare-metal stents (38, 101). Additionally, Tang et al. developed a novel drug-coated balloon incorporating lipophilic drug-loaded nanomotors. This system harnesses excess ROS in atherosclerotic lesions to react with arginine, generating NO as a propulsion mechanism. This enhances the local retention and tissue penetration of PTX and triphenylphosphonium (TPP), while concurrently improving endothelial function, reducing oxidative stress, and inhibiting cell proliferation and neointimal formation, achieving a multifaceted therapeutic effect (102).

Rapamycin-based drugs bind to FK506-binding protein 12 (FKBP12), selectively inhibiting the mTORC1. This blockade halts VSMCs progression from the G1 to S phase of the cell cycle, suppresses synthetic phenotypic switching, and attenuates ECM synthesis and secretion (100). Ryu et al. investigated the combined local administration of sirolimus and rosuvastatin for inhibiting IH following CABG, integrating network pharmacology and a rabbit abdominal aortic balloon injury model. Their analysis identified 96 common drug targets relevant to CABG, suggesting potential synergism through the IL-6/STAT3 pathway (acute phase) and AKT/MMP9 pathway (chronic phase). *In vivo* validation showed that the combination significantly reduced neointimal thickness (406.74 ± 196.18 μm vs. 947.64 ± 429.14 μm) and the intima-to-media ratio (13.15 ± 11.18% vs. 31.73 ± 7.16%) at 4 weeks post-surgery, along with downregulation of IL-6, TNF-α, STAT3, AKT/mTOR, and NF-κB signaling proteins (103). Similarly, Bai et al. employed a hyaluronic acid/sodium alginate (HA/SA)-based hydrogel to deliver poly(lactic-co-glycolic acid) (PLGA)-encapsulated rapamycin nanoparticles. In a rat aortic guidewire injury model, this perivascular delivery system significantly reduced neointimal thickness (*p* = 0.0009), inflammatory cell infiltration (CD68^+^ macrophages, *p* = 0.0012; CD3^+^ T cells, *p* = 0.0011), phosphorylated mTOR (*p* = 0.0019), and PCNA^+^ proliferating cells (*p* = 0.0028) at 21 days post-injury, while preserving endothelial-specific markers Ephrin-B2 and Delta-like ligand 4 (Dll-4) (104).

These studies have laid a solid theoretical foundation and offered promising therapeutic directions for the prevention and treatment of vascular restenosis and IH. As nanocarrier systems continue to be optimized, and as research deepens into drug release kinetics, targeted delivery, and long-term retention in diseased tissues, strategies for managing IH are shifting from traditional passive inhibition of cell proliferation to active modulation of the vascular microenvironment. This shift provides a scientific basis for the advancement of cardiovascular disease therapies.

### 6.4 Gene therapy

Gene therapy represents a frontier in the treatment of IH. It fundamentally reconstructs the gene expression network of VSMCs by upregulating or downregulating pivotal genes, thereby reversing their pathological phenotype. This approach offers the potential for sustained inhibition and even regression of IH.

Recent studies have demonstrated the therapeutic potential of modulating key molecular targets involved in this process. For example, specific knockout of interleukin enhancer-binding factor 3 (ILF3) in VSMCs promotes the degradation of high mobility group box 1 (HMGB1) mRNA and inhibits the phosphorylation of signal transducer and activator of transcription 3 (STAT3). These effects significantly suppress PDGF-BB-induced phenotypic switching, proliferation, and migration of VSMCs, ultimately reducing neointimal formation (105). In terms of delivery systems, solanaceous plant-derived exosome-like nanoparticles (SL-ELNs) have shown efficient gene delivery capability. These nanoparticles transport miR-164a/b-5p, which downregulates the mRNA expression of Kelch-like ECH-associated protein 1 (Keap1), promotes nuclear translocation of nuclear factor erythroid 2–related factor 2 (Nrf2), and activates the transcription of antioxidant genes. As a result, SL-ELNs effectively inhibit PDGF-BB-induced VSMCs proliferation, migration, and phenotypic switching, significantly reducing IH in vascular injury models (106). Zhao et al. developed an adventitial delivery system based on an injectable RAD peptide hydrogel—composed of self-assembling arginine-alanine-aspartic acid repeat sequences—to achieve sustained release of a miR-145-5p agomir. *In vitro* studies confirmed continuous release and efficient transfection of miR-145-5p, enhancing the expression of contractile phenotype markers and inhibiting VSMCs migration. *In vivo* experiments further demonstrated that the system prolongs miR-145-5p retention at the injury site, suppresses proliferative VSMCs via downregulation of Krüppel-like factor 4 (KLF4), promotes endothelial regeneration, reduces macrophage infiltration, inhibits neointimal formation, and facilitates ECM remodeling (107).

These advances suggest that, with a deeper understanding of the molecular mechanisms driving VSMCs phenotypic switching and the development of innovative gene delivery platforms, the integration of gene therapy with localized intervention strategies may represent a critical breakthrough in cardiovascular disease treatment. As delivery systems continue to improve in precision and safety, gene therapy is poised to offer novel therapeutic options for patients with vascular remodeling disorders.

### 6.5 Venous external support (VEST)

VEST provides a biomechanical solution to prevent postoperative IH in SVGs. By reducing wall tension, limiting lumen dilation and irregular remodeling, external stents can optimize hemodynamic conditions and shear stress, thereby mitigating IH and preserving the structural integrity and long-term patency of SVGs (108).

The VEST III multicenter randomized trial (*n* = 184) by Taggart et al. evaluated the 2-year outcomes of SVGs supported by VEST compared with unsupported autologous grafts. The results showed no significant difference in overall graft patency between the two groups (78.3% vs. 82.2%, *P* = 0.43). However, the proportion of completely patent grafts (Fitzgibbon grade I) was significantly higher in the VEST group (66.7% vs. 54.9%, OR = 2.02, *P* = 0.03). Intravascular ultrasound (IVUS) further confirmed a 22.5% reduction in intimal area (3.07 vs. 3.96 mm^2^, *P* < 0.001) and a 23.5% reduction in intimal thickness (0.26 vs. 0.34 mm, *P* < 0.001). While the VEST did not significantly enhance short-term patency, it effectively inhibited IH and improved structural preservation of the graft. Long-term clinical benefits, however, remain to be confirmed through extended follow-up (109). Another randomized within-patient controlled trial (*n* = 224) assessed the 1-year safety and efficacy of VEST. The study found no statistically significant differences in IH or safety endpoints between the VEST and control groups. However, a higher-than-expected rate of SVG occlusion limited the ability to conduct IVUS in some grafts, requiring data imputation that may have compromised the reliability of the results. Nevertheless, in the subgroup with complete IVUS data, the VEST group exhibited significantly less IH than controls (110). These findings highlight the importance of early postoperative management to prevent SVG occlusion and suggest a need for continued investigation into the long-term benefits of VEST in improving graft patency and patient prognosis.

Despite receiving early Conformité Européenne approval in Europe, the use of VEST devices in SVG revascularization is not currently recommended by either the 2018 European Society of Cardiology/European Association for Cardio-Thoracic Surgery (ESC/EACTS) guidelines or the 2021 American College of Cardiology/American Heart Association (ACC/AHA) revascularization guidelines (111). The clinical utility of VEST remains controversial due to the lack of robust evidence linking it to definitive clinical endpoints. Therefore, further high-quality, long-term studies are required to determine its efficacy and role in clinical practice.

### 6.6 Clinical and perioperative strategies

In addition to molecular, pharmacological, and device-based approaches, several modifiable clinical and perioperative factors substantially influence the development of IH and long-term graft patency. Effective lipid management—particularly with statins—enhances endothelial function, reduces vascular inflammation, and stabilizes vascular remodeling. Smoking cessation is essential, as tobacco exposure exacerbates oxidative stress and endothelial injury, thereby accelerating VSMCs activation and IH progression. Antiplatelet therapy, including aspirin or dual antiplatelet therapy when indicated, mitigates platelet-driven inflammation and early thrombotic events that predispose to maladaptive healing. Optimal control of systemic cardiovascular risk factors—such as diabetes, hypertension, dyslipidemia, and obesity—further contributes to a favorable vascular milieu and attenuates chronic inflammatory signaling implicated in VSMCs phenotypic switching. From a surgical standpoint, perioperative management principles are equally critical. Gentle conduit handling, minimization of mechanical manipulation, appropriate regulation of intraluminal pressure during preparation, reduction of warm ischemia time, and maintenance of physiological anastomotic geometry all help limit the initial endothelial injury that initiates IH (6, 7, 13, 83, 112). These clinical and perioperative measures complement targeted interventions and collectively support improved graft maturation and long-term patency. Collectively, they optimize the biological environment in which graft remodeling occurs and remain consistent with the broader therapeutic framework depicted in Figure 3.

## 7 Conclusions and outlook

CABG remains a critical revascularization strategy for patients with complex coronary artery disease, with the great saphenous vein widely utilized as a graft conduit. However, the long-term patency of vein grafts remains suboptimal, primarily due to postoperative IH and accelerated atherosclerosis. At the core of this pathological process lies the phenotypic switching of VSMCs. During arterial adaptation, VSMCs shift from a contractile to a synthetic phenotype, exhibiting increased migration, proliferation, and abnormal secretion of inflammatory mediators and ECM components. This review systematically summarizes the developmental stages and molecular mechanisms underlying VGF, with an emphasis on the signaling pathways that regulate VSMCs phenotypic switching. Key signaling axes include PDGF-BB, TGF-β, MAPK, mTOR, and NF-κB, as well as the modulatory roles of non-coding RNAs. This article highlights that endothelial dysfunction, inflammatory microenvironments, and altered hemodynamics jointly contribute to pathological VSMCs activation, thereby promoting neointimal formation and vascular remodeling. Current strategies to prevent and manage VGF include optimization of surgical techniques (e.g., the no-touch vein harvesting method and improved vein storage solutions), pharmacological interventions (e.g., paclitaxel and rapamycin), gene therapy, and the use of venous external stents. The no-touch technique reduces postoperative occlusion by preserving the structural integrity of the vein's adventitia. Drug-coated devices and adventitial drug delivery systems locally release anti-proliferative agents to inhibit VSMCs phenotypic switching and neointima formation. Gene therapy enables targeted regulation of VSMCs behavior by delivering specific non-coding RNAs or modulating key signaling molecules. Meanwhile, venous external stents aim to modulate hemodynamic forces and vascular remodeling, though their long-term efficacy remains under investigation. Complementing targeted interventions, modifiable clinical and perioperative factors—including lipid management, antiplatelet therapy, cardiovascular risk control, smoking cessation, and careful conduit handling—further promote graft maturation and long-term patency by fostering a favorable vascular milieu.

Despite meaningful progress in mechanistic research and clinical interventions, several challenges remain unresolved. First, the regulatory network governing VSMCs phenotypic switching is highly complex, involving extensive cross-talk between signaling pathways. Future studies should leverage single-cell sequencing and spatial transcriptomics to dissect the heterogeneity and dynamic behavior of VSMCs subpopulations during disease progression. Second, although drug-eluting devices can effectively suppress IH, their long-term use may impair endothelial healing. Therefore, the development of dual-functional delivery systems that simultaneously inhibit proliferation and promote endothelialization is of great significance. In the context of gene therapy, challenges related to delivery efficiency and tissue specificity persist. Advancing delivery platforms—such as exosome-like nanoparticles and injectable hydrogels—will be key to improving the targeting and biocompatibility of gene-based treatments. Moreover, the long-term clinical benefits of venous external stents remain controversial, necessitating further exploration into their potential synergistic application with pharmacological or gene-based interventions, such as using biodegradable stents as local carriers for sustained drug or gene release to achieve both mechanical support and molecular modulation.

In conclusion, elucidating the molecular mechanisms underlying VSMCs phenotypic switching, in combination with advances in targeted delivery technologies and personalized treatment approaches, holds promise for enhancing long-term venous graft patency and improving clinical outcomes in patients undergoing CABG.
